# Treatment of tuberculosis infection among immigrants in southern New Brunswick, Canada: A cross-sectional study

**DOI:** 10.14745/ccdr.v51i101112a03

**Published:** 2025-12-12

**Authors:** Isdore Chola Shamputa, Duyen Thi Kim Nguyen, Hope Mackenzie, Derek J Gaudet, Alicia Harquail, Kim Barker, Duncan Webster

**Affiliations:** 1 Department of Nursing and Health Sciences, University of New Brunswick, Saint John, NB; 2 Department of Health, Government of New Brunswick, Saint John, NB; 3 Faculty of Business, University of New Brunswick, Saint John, NB; 4 Microbiology Laboratory, Saint John Regional Hospital, Saint John, NB; 5 Department of Psychology, University of New Brunswick, Saint John, NB; 6 Dalhousie Medicine New Brunswick, Dalhousie University, Saint John, NB; 7 Division of Medical Microbiology, Department of Laboratory Medicine, Saint John Regional Hospital, Saint John, NB; 8 Division of Infectious Diseases, Department of Medicine, Saint John Regional Hospital, Saint John, NB

**Keywords:** tuberculosis infection, treatment, management factors, immigrants, southern New Brunswick

## Abstract

**Background:**

Treating individuals with tuberculosis (TB) infection (TBI) is an important aspect of the global strategy to eliminate TB as a public health problem, as it would help reduce the pool of individuals with TBI who are at risk of developing TB disease (TBD). Understanding factors that impact effective management of patients with TBI is helpful in informing policy.

**Objective:**

To assess the proportion of immigrants with TBI accepting and completing TB preventive treatment (TPT), variables potentially related to accepting and completing TPT were examined and healthcare provider (HCP)-related factors that impact TBI management were identified.

**Methods:**

Tuberculosis preventive treatment was offered to TBI-positive immigrants without a history or treatment of TBD from a pilot TBI screening study conducted in southern New Brunswick, Canada between November 2021 and November 2023. Tuberculosis preventive treatment acceptance and completion rates were calculated, and the HCP completed a questionnaire to identify factors that affected TBI management. Participant characteristics were summarized using descriptive statistics, while Fisher’s exact tests were conducted to test for independence between demographics and treatment acceptance and completion. The HCP questionnaire data were analyzed using thematic analysis.

**Results:**

Of the 49 participants who screened positive for TBI, 11 (22.4%) were lost to follow-up prior to being assessed and offered TPT and 38 (77.6%) were offered TPT, of whom 3 (7.9%) declined, 35 (92.1%) accepted and initiated TPT, and 30 (85.7%) completed treatment. Treatment acceptance and completion were found to be independent from the participant demographics examined. Thematic analysis revealed five emerging themes regarding the management of TBI participants (i.e., supports, collaboration, communication, time, and satisfaction).

**Conclusion:**

This study demonstrates the feasibility of treating TBI in immigrants and highlights HCP-related factors that impact the management of TBI among immigrants in southern New Brunswick. Our findings may assist programs aimed at improving TBI screening and treatment.

## Introduction

Tuberculosis (TB) is the leading cause of death from a single infectious agent, with 10.8 million reported cases and 1.25 million fatalities globally in 2023 ((1)). A major concern is that approximately one-quarter of the world’s population is estimated to have TB infection (TBI) ((2)), an asymptomatic condition that can lead to TB disease (TBD) in 5%–10% of those infected ((3)).

In 2001, Canada adopted the Stop TB Partnership goal of eliminating TB as a public health threat by 2030 ((4)). In 2014, Canada signed the World Health Organization (WHO)’s *Towards tuberculosis elimination: an action framework for low-incidence countries*, which set a pre-elimination target of fewer than one TBD case per 100,000 people by 2035 and full elimination, defined as fewer than 0.1 cases per 100,000 people per year, by 2050 ((5)). Although Canada reported a low TBD incidence rate of 5.1 cases per 100,000 people in 2022 (6), to achieve the full TB elimination target by 2050, the country needs to reduce its annual TBD incidence by more than 10% ((5)). The TBD incidence in Canada decreased from the 1990s to the early 2000s, was maintained at that lower rate for over a decade, and then started rising steadily from 2014 onward ((6,7)).

In New Brunswick (NB), an Atlantic Canadian province, the incidence of TBD has consistently remained below the national average, though it has shown a slight upward trend, rising from 0.4 cases per 100,000 people in 2013 to two cases per 100,000 population in 2022 ((6)).

This increase parallels the province’s population growth, which is largely driven by immigration ((8)), and is widely believed to stem from the reactivation of TBI acquired before arriving in Canada ((9,10)). To counter this more recent trend, it is essential to adopt innovative strategies, such as providing routine screening and treatment of TBI among at-risk populations, as this could help lower the number of people at risk of developing TBD and help with the country’s commitment to meeting the WHO’s goal of reaching less than one TBD case per 100,000 population by 2035 ((5)). Individuals immigrating to Canada are required to undergo immigration medical examinations (IMEs) pre-arrival. These examinations include a chest X-ray to screen for TBD in everyone aged 11 and older, along with TBI screening for specific high-risk populations ((11)). In 2022, 76.2% of TBD cases in Canada ((6)) as well as all TBD cases in NB in 2022 (n=17) and 2023 (n=14) were among immigrants (*personal communication, Public Health Agency of Canada Tuberculosis Task Force meeting, November 26–27, 2024*), yet routine screening and treatment for TBI among immigrants is not widely available ((9)).

There is a dearth of data on rates of acceptance and completion of TBI treatment in Canada, as well as factors that affect the management of individuals with TBI. Previous studies on TBI screening and treatment in Canada were conducted among refugees at refugee health centres ((12–18)). Two of these studies also examined healthcare worker-related factors associated with TBI management ((12,13)).

This study seeks to contribute to the body of knowledge by determining the acceptance and completion rates of TBI treatment among eligible immigrants of all immigration streams in southern NB, Canada, a region with a rising number of immigrants from TB-endemic areas, albeit without an immigrant health clinic. Furthermore, this study seeks to examine variables potentially related to accepting and completing TB preventive treatment (TPT) and examine healthcare provider (HCP)-related factors that impact the management of individuals with TBI. This study was part of a pilot TBI screening program for immigrants residing in southern NB, which offered TPT following clinical assessment among those who screened positive ((19)).

## Methods

Study participants, recruited consecutively, were ≥19 years of age, resided in southern NB, Canada, and born in a country with a TB incidence of ≥40 cases per 100,000 people or were considered at high risk of TBI by an HCP and were referred for TBI screening in a study conducted between November 2021 and November 2023 ((19)). The study was conducted in southern NB partly because there is no dedicated refugee or immigrant health clinic in the area. This setting is of further importance, given the increased number of immigrants arriving in the area from TB-endemic countries. Tuberculosis infection screening was performed using the interferon-gamma release assay (IGRA), (QuantiFERON-TB Gold Plus, Qiagen, Germantown, Maryland, United States). To be eligible for this study, participants were required to have screened positive for TBI without prior TPT and have no history or treatment for TBD upon clinical assessment.

### Tuberculosis infection treatment procedures

Upon clinical assessment, participants diagnosed with TBI who consented to TPT and were without contra-indications were offered first-line TBI treatment regimens by the treating HCP, as recommended by the Canadian Tuberculosis Standards: 1) rifampicin (4R) – four-month duration with self-administered daily dosing; or 2) isoniazid/rifapentine (3HP) – three-month duration with 12 once per weekly doses observed and monitored at the participant’s community pharmacy ((20)). All medications provided to participants were covered under the provincial health plan and were dispensed by community pharmacies. Treatment was initiated and was accompanied by routine monitoring blood work to assess for adverse effects, as per Canadian Tuberculosis Standards recommendations ((20)). The decision to move forward with TPT was made in consultation with the patient following counselling on the natural history of TB and individualized risk for reactivation to TBD. Patients were counselled by the treating HCP. If the patient expressed interest in moving forward with TPT, they were offered options for treatment with a focus on 4R and 3HP. Risks and benefits were discussed. In each case, if English was not the primary language of the patient or if there was a preference to use an alternate language, a translator was used to allow for back-and-forth dialogue in real-time to ensure the patient was able to receive the needed information and ask all desired questions to ensure understanding around the risks and benefits of TPT. Interpreters were also available for follow-up. The infectious disease physician, public health nurses, community nurses, local pharmacies, and community organizations provided screening and treatment support based on participants’ needs.

Treatment was considered complete when participants reported taking all prescribed doses within the designated timeframe. Treatment adherence was corroborated by reviewing the dispensing history of the community pharmacy. Dropout rates and the reasons for dropping out at each stage were recorded. Participants who declined treatment were counselled on the natural history of TB by the HCPs and were encouraged to undergo annual chest X-rays for two years from their arrival date in Canada. Local immigrant-serving organizations, namely the YMCA of Greater Saint John and the Saint John Newcomer Centre, provided substantial support to study participants and HCPs throughout the treatment process. This support included assistance with navigating the healthcare system through the clinical encounters, diagnostic imaging, and scheduled phlebotomy, as well as community guidance with pharmacy services and refills when needed. The organizations also served as a communication conduit between the patient and the healthcare team and public health. This was critical in navigating a multitude of logistical challenges and assisting with scheduling and rescheduling appointments.

## Data collection and analyses

### Treatment acceptance and completion

Demographic data and treatment variables of participants, including age, sex, gender, year of arrival to Canada, type of visa used on the participants’ initial entry to Canada, country of birth, and TB incidence in the country of birth were collected during the recruitment process ((19)). In addition, a history of the country of repatriation prior to arrival in Canada was obtained. Other countries and regions where the participants had lived were also noted. As a component of the clinical history, details pertaining to the precise region of the countries where study participants had lived in addition to a timeline of migration were gathered and documented. Country of birth was categorized into one of six WHO regions ((21)). Tuberculosis infection positivity was reported using defined WHO incidence rate categories ((22)). The type of treatment each participant received, reasons for loss to follow-up, and not initiating or completing TPT were documented. Proportions for each step of the treatment cascade were calculated and reasons for loss to follow-up and not initiating or completing treatment were recorded. Participant demographic variables were summarized using descriptive statistics. To test for independence between each of the demographic variables listed in **Table 1** and the acceptance and completion variables, the dataset was imported into RStudio (version 2024.12.0.467). To account for the small sample size and expected cell counts in the contingency tables being less than five, in some cases, Fisher’s exact tests were used. Analyses were conducted using the ‘fisher.test()’ command in RStudio (version 2024.12.0.467). To address the potential issue of multiple comparisons, a more stringent alpha criterion of 0.01 was applied.

**Table 1:** Demographics and tuberculosis infection treatment acceptance and completion with Fisher’s exact test

Demographic characteristics	Treatment acceptance (n)		*p*-value	Treatment completion (n)		*p*-value
	Yes	No		Completed	Not completed	
**Sex**						
Male	14	1	-	12	2	-
Female	21	2	-	18	3	-
Total	35	3	1.00	30	5	1.00
**Year of arrival in Canada**						
2001	1	0	-	1	0	-
2014	1	0	-	1	0	-
2016	0	1	-	0	0	-
2018	1	0	-	1	0	-
2019	3	0	-	3	0	-
2021	5	0	-	4	1	-
2022	8	2	-	7	1	-
2023	16	0	-	13	3	-
Total	35	3	0.084	30	5	1.00
**Visa type on initial entry to Canada**						
Family reunion	1	0	-	1	0	-
Visitor	1	0	-	1	0	-
Permanent resident	4	1	-	3	1	-
Study	7	0	-	7	0	-
Government-assisted refugee	22	2	-	18	4	-
Total	35	3	0.531	30	5	0.610
**Country of birth by WHO region**						
Western Pacific	5	0	-	5	0	-
Americas	3	1	-	2	1	-
Africa	10	1	-	8	2	-
South-East Asia	2	0	-	2	0	-
Eastern Mediterranean	15	1	-	13	2	-
Total	35	3	0.635	30	5	0.746
**TB incidence by country of birth (per 100,000 population)^a^**						
Lower–moderate (10‒9)	3	1	-	3	0	-
Upper‒moderate (50‒99)	9	0	-	6	3	-
Endemic (100‒299)	15	1	-	13	2	-
Highly endemic (300‒499)	4	1	-	4	0	-
Severely endemic (≥500)	4	0	-	4	0	-
Total	35	3	0.331	30	5	0.528
**Age range, years**						
19‒24	3	0	-	3	0	-
25‒34	7	0	-	4	3	-
35‒44	18	1	-	16	2	-
45‒54	5	0	-	5	0	-
55‒64	2	1	-	2	0	-
65 and older	0	1	-	0	0	-
Total	35	3	0.108	30	5	0.382
**Treatment regimen^b^**						
Rifampicin (4 months)	-		-	23	4	-
Isoniazid and rifapentine (3 months)	-		-	6	1	-
Intermittent isoniazid (9 months)	-		-	1	0	-
Total	-		-	30	5	1.00

Abbreviations: TB, tuberculosis; WHO, World Health Organization; -, not applicable

^a^ World Health Organization. WHO global lists of countries for tuberculosis (TB), TB/HIV and multidrug/rifampicin-resistant TB (MDR/RR-TB), 2021–2025: Background document. Geneva, CH: WHO; 2021. ((22))

^b^ Adapted from Alvarez GG, Pease C, Menzies D. Chapter 6: Tuberculosis preventive treatment in adults. Can J Respir Crit Care Sleep Med 2022;6(sup1):77–86. ((20))

### Healthcare provider survey

At study completion, the HCP completed a pilot-tested survey regarding their experiences in managing TBI positive participants ((23)), **Appendix Table A1**. The survey was developed by the research team and pilot-tested with four HCPs to evaluate clarity, coherence, and completion time. The survey was revised based on feedback received, which addressed the wording and sequencing of both the initial and follow-up questions. The survey consisted of 10 dichotomous questions, nine of which had open-ended follow-up questions to gather additional clarification if the HCP answered “yes” to any of the dichotomous questions. The questions aimed to collect information about the HCPs’ role, the adequacy of tools needed to support and care for patients during both initial and follow-up visits, barriers in arranging follow-up tests, and the helpful aspects of the care process throughout the pilot screening program. The survey also sought to determine the average time spent per patient visit, the total time dedicated to patient management (including administrative tasks) and any final comments the HCPs wished to provide. The survey was completed virtually through an email containing an attachment sent by a research team member. The HCP survey data were analyzed using thematic analysis to identify recurring patterns and emerging themes to gain a better understanding of the HCP’s views, opinions, and experiences ((24)).

### Ethical approval

The main component of this sub-study was approved by the Horizon Health Network (file #: RS 2021-3046) and the University of New Brunswick (file #: 033-2021) Research Ethics Boards.

## Results

### Participant characteristics

This study included 49 participants who screened positive for TBI ((16)). Participants consisted of 21 males and 28 females, with a mean age of 40.2 years (range: 19–67). Most participants (85.7%, n=42) arrived in Canada between 2021 and 2023 and were predominantly government-assisted refugees (71.4%, n=35) from the Eastern Mediterranean (53.1%, n=26) WHO region. Other participants held permanent resident (n=5) and study (n=7) visas. Additionally, two participants held a family reunion and visitor visas, respectively, when they first arrived in the country. Approximately half (51%, n=25) of participants were born in a TB-endemic country, defined as TB incidence of 100–299 cases per 100,000 people. A detailed list of participant characteristics is presented in **Table 2**. Two HCPs on the research team, consisting of an infectious disease physician and a nurse practitioner, managed participants throughout their treatment process.

**Table 2:** Descriptive statistics of participants who were eligible for tuberculosis preventive treatment, (N=49)

Variables	Male (n=21)	Female (n=28)	N=49
**Age range, years**			
19–24	1	2	3
25–34	3	8	11
35–44	11	11	22
45–54	4	4	8
55–64	1	3	4
65 and older	1	0	1
**Year of arrival in Canada**			
2001	1	0	1
2014	0	1	1
2016	0	1	1
2018	1	0	1
2019	3	0	3
2021	1	4	5
2022	8	10	18
2023	7	12	19
**Visa type on initial entry to Canada**			
Family reunion	0	1	1
Visitor	1	0	1
Permanent resident	1	4	5
Study	2	5	7
Government-assisted refugee	17	18	35
**Country of birth by WHO region**			
Western Pacific	3	3	6
Americas	1	3	4
Africa	2	9	11
South-East Asia	0	2	2
Europe	0	0	0
Eastern Mediterranean	15	11	26
**TB incidence by country of birth (per 100,000 population)^a^**			
Low (<5)	0	0	0
Lower-moderate (10–49)	2	4	6
Upper-moderate (50–99)	4	5	9
Endemic (100–299)	14	11	25
Highly endemic (300–499)	0	5	5
Severely endemic (≥500)	1	3	4
**Primary healthcare provider (doctor/nurse practitioner) in Canada**			
No	20	27	47
Yes	1	1	2

Abbreviations: TB, tuberculosis; WHO, World Health Organization

^a^ World Health Organization incidence

### Tuberculosis infection treatment outcome

**Figure 1:**
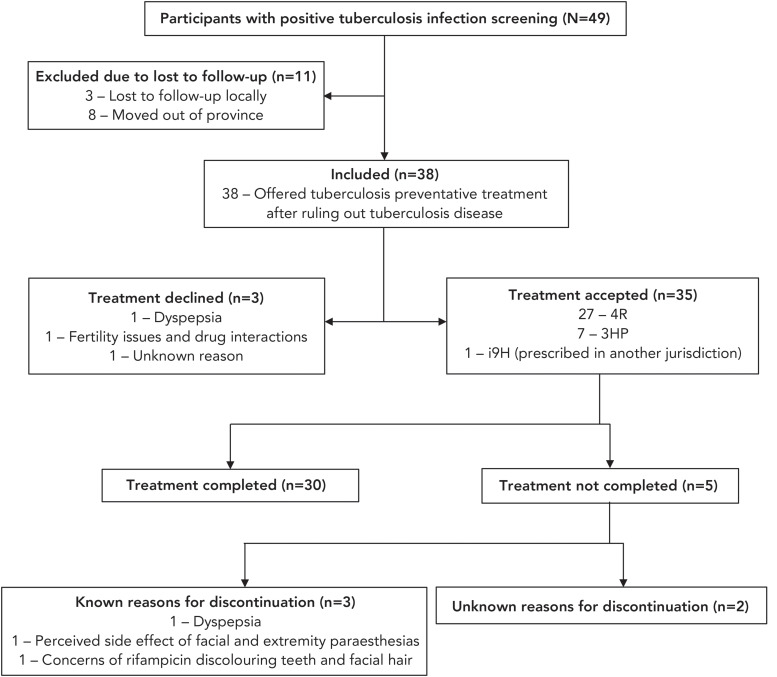
Flow chart of immigrants with tuberculosis infection eligible for tuberculosis preventive treatment in southern New Brunswick, Canada, (N=49) Abbreviations: 3HP, isoniazid/rifapentine; 4R, rifampicin; i9H, intermittent isoniazid

Of the 49 participants who screened positive, 11 were lost to follow-up prior to the clinical assessment. Of these, three were lost to follow-up in the local setting, while eight moved out of province before they could be assessed and offered TPT. The remaining 38 participants (77.6%), who underwent clinical assessment, were offered TPT after ruling out TBD and confirming TBI with no prior TPT. Among these 38 participants, following counselling on the natural history of TB and the risks and benefits of treatment, 35 (92.1%) accepted TPT, of whom 30 (85.7%) completed treatment and five (14.3%) did not complete TPT. Notably, while the treatment completion rate across most defined age groups was generally high (88.9%–100%), only four out of seven (57.1%) participants in the 25–34 age group completed their treatment. Of the five (14.3%) participants who did not complete treatment, three discontinued due to dyspepsia, perceived side effects such as facial and extremity paresthesias, and concerns about rifampicin causing discoloration of teeth and facial hair, respectively. The reasons for treatment discontinuation in the remaining two participants are unknown. A more detailed account of the participants included in this study is provided in **Figure 1** and Table 1.

The infectious disease physician oversaw the care of all but two participants—one was cared for by the nurse practitioner, and another was managed by an HCP in a province/territory where they had relocated following the clinical assessment. The participant (n=1, 2.9%) who relocated to another province/territory following initial clinical assessment and before initiating TPT was prescribed twice weekly intermittent isoniazid treatment for nine months by the provincial/territorial public health program in the new location; this patient remained in contact with the study physician throughout the duration of the study and was included in this study analysis (Figure 1, Table 1). Treatment adherence was assessed through patient reports and follow-up by the treating physician who tracked patient-reported adherence in combination with dispensing history from the community pharmacy to ensure reported adherence correlated with the timing of refill dispensing.

Assistance from immigrant-serving organizations was helpful in establishing and maintaining successful contact for several participants before and during treatment, including three participants who needed to restart treatment after an interruption. Among the eight participants who moved out of the province before clinical assessment and initiating treatment, four (50%) were from the same family. One participant obtained a new HCP in their new province, and a letter was dictated to their new HCP detailing the positive IGRA.

Fisher’s exact tests were run to determine whether treatment acceptance and completion were dependent on sex, arrival year, visa type, country and TB incidence of the country of birth by WHO region, and age. An assessment was also conducted on whether treatment completion was dependent on treatment regimen. None of the Fisher’s exact tests for independence yielded statistically significant results, indicating that treatment acceptance and completion were independent of the examined variables. The *p*-values for each test are provided in Table 1.

### Thematic analysis

Thematic analysis was used to analyze qualitative survey data. The infectious disease physician completed the HCP survey, and thematic analysis of the feedback revealed five emerging themes that affected the management of TBI among immigrants in southern NB. Themes and supporting statements are listed in **Table 3** and below:

**Table 3:** Themes and supporting statements

Themes	Illustrative quotes
Support system for participants	“Overall, the patient support required was available.” “The YMCA health liaison and the local newcomer associations were very supportive.”
	“For those newcomers who had recently arrived in New Brunswick, a navigator to assist with moving through the medical system would be very helpful.”
Collaboration and support mechanisms	“The YMCA and newcomers associations were a big help.”
	“Community pharmacy partners were also incredibly valuable.”
	“Clear Canadian guidelines were instrumental.”
	“The use of the IGRA allowed for clear documentation of objective results in all cases.”
Communication and follow-up challenges	“Follow-up was challenging as some individuals were hard to reach.”
	“Many individuals did not understand the concept of a refill.”
	“Setting up the recommended one-month bloodwork was sometimes challenging.”
	“Having written or typed information on TB and treatment in various languages would have helped with the first visit in many cases.” “Patient information in various languages would improve care.”
Time allocation in participant care	“I spent one hour for the initial visit.”
	“Roughly 15 minutes per follow-up visit.”
	“I spent an average of approximately two hours per patient overall.”
Overall satisfaction with care	“The preventive treatment was also well received, overall.”

Abbreviations: IGRA, interferon-gamma release assay; TB, tuberculosis

1) Support systems for participants: During the study, it was reported that resource adequacy impacted participant care, highlighting factors such as availability of support and need for participant navigators.

2) Collaboration and support mechanisms: This theme revealed the invaluable support provided by immigrant-serving organizations and community pharmacy partnerships in caring for immigrants with TBI. As well, clear national TPT guidelines and objective diagnostic tools allowed the HCP to work efficiently and effectively.

3) Communication and follow-up challenges: The HCP experienced challenges when trying to reach participants and when communicating during the cascade of care. Due to a lack of multilingual resources at certain points in the cascade of care, the HCP took extra precaution to help ensure participants understood the follow-up procedures, with particular reference to bloodwork coordination.

4) Time allocation in participant care: It was identified that more time during appointments were required for newcomer participants, due to, for example, language barriers, assisting with healthcare navigation, answering questions and educating participants regarding treatment procedures, side effects.

5) Satisfaction with care: Finally, the HCP reported that participants generally responded positively to TPT and care. For example, participants expressed appreciation with regards to receiving information pertaining to TBI and clinical support and follow-up during the course of TPT. Participants also expressed feeling positive upon completion of treatment.

## Discussion

This study is the first to assess TPT acceptance and completion rates in Atlantic Canada and the third in the country to investigate HCP-related factors affecting the management of individuals on TPT ((12,13)). The 92.1% TBI treatment acceptance and 85.7% treatment completion rates reported in this study are concordant with rates reported in Canada, which range from 75.0% to 93.4%, and 49.0% to 100%, respectively. The HCP’s feedback identified key factors that could enhance TBI management for immigrants. The rates reported herein are promising, especially considering adherence to TPT is often low ((25,26)). Treatment acceptance or completion was not found to be associated with any of the examined demographic and treatment variables. Although we had an additional variable (TB incidence category) and one variable (age) was categorized differently, our results were largely consistent with the findings of Harwood-Johnson *et al.* ((14)) but differed in one respect. Whereas age was found to have been associated with acceptance in their study, here it was not. Harwood-Johnson *et al.* ((14)) reasoned that the association found in their study was likely due to the dichotomous age categories of those older than 18 years and those younger than 18 years, where those less than the age of majority would have decisions made for them by a legal guardian. Participants in the current study were all above the age of majority. The findings of this study, together with those of Harwood-Johnson *et al.*, suggest that age might only be a factor in treatment acceptance when also considering those under the age of majority.

The TPT acceptance and completion rates in this study surpass those reported in a recent global systematic review and meta-analysis of migrants, which indicated TPT initiation and completion rates of 69% and 74%, respectively ((27)), but within rates reported in Canada ((12–18)). Individualizing treatment options in the current study was seen as an opportunity to enhance completion rates. Participants were given the option to choose either the 4R or 3HP regimens or consider no medical therapy with clinical and radiological follow-up offered for up to two years. The majority of participants preferred to move forward with the 4R regimen. Although this treatment option is longer in duration, it enabled participants to dose daily in their own setting. In contrast, the shorter 3HP regimen required observed dosing at their community pharmacy once per week.

Consistent with reports from prior studies ((12,25,28)), barriers such as missed appointments, difficulties rescheduling, and geographic mobility significantly contributed to attrition throughout the treatment cascade in the current study. Missed appointments remain a persistent challenge, particularly among new arrivals who may struggle with navigating the healthcare system and adhering to scheduled appointments. Immigrant-serving organizations helped minimize barriers by providing essential information and support to assist individuals manage healthcare appointments more effectively. However, these organizations remain with limited resources and a lack of stable funding.

In this study, moving out of province primarily impacted government-assisted refugees recruited shortly after their arrival during their post-arrival health assessment (PAHA). Intentions to relocate to other provinces were often not communicated to their navigators or study investigators until after the fact. To assist in diminishing this concern, it may be beneficial to inquire about potential medium- to long-term settlement plans during the PAHA. Alternatively, delaying invitations for several weeks could allow for better assessment of stability. However, this latter approach may lead to even greater attrition with loss of access to language translation services necessary for many government-assisted refugees who often only had temporary residences and frequently no cell phones or email addresses for communication. A third option would be to wait until participants have access to primary HCPs before conducting assessments. However, this could present additional challenges considering only two of the participants in this study had access to primary HCPs. The time it takes for individuals to be assigned a primary HCP can vary significantly in our context. Furthermore, routine treatment of TBI through primary HCPs is not generally offered in this setting.

While relocating out of province has often been linked to disruptions in care, effective collaboration among HCPs across provincial/territorial lines can help maintain continuity of care. This was demonstrated in the current study with two participants. In one case, the HCP coordinated with the participant’s new HCP in the other province, ensuring a smooth transition for the participant’s treatment. In another instance, the HCP provided a detailed note to the receiving HCP in another province, which ensured seamless transfer of care for the participant.

Although medication side effects have been noted in other studies as a significant reason for participants discontinuing treatment ((29–32)), their impact in this study was minimal, as only three participants cited this as the plausible reason for discontinuing treatment. Close follow-up to assist with managing minor adverse effects and enhanced education of participants on the importance of completing treatment may help address this limitation.

In this study, coordination between HCPs, community organizations, and public health entities was essential for effective TBI management. This finding aligns with current public health strategies that emphasize a multidisciplinary approach to TB care, promoting better health outcomes through shared responsibility among various stakeholders ((33)). There were several participants who appeared to not fully understand the need of adhering to treatment or the potential consequences of non-compliance. Conducting participant education initiatives, offering culturally sensitive materials in the predominant languages spoken by immigrants, and follow-up reminders may help mitigate these challenges.

Limited access to HCPs noted in other studies was not a concern in this study, thanks to dedicated HCPs involved in the research, even though nearly all participants were primarily cared for by one HCP ((13)). Although it is unlikely to represent the situation in regular clinical settings, the use of a single HCP resulted in less variability in patient management, a more comprehensive understanding of each patient’s needs, and improved data accuracy. Feedback from the HCP suggested that some participants required more time to be provided with adequate care. Increased staffing levels throughout the cascade of care and continuous quality improvement measures that focus on enhancing the participant experience may improve TBI treatment acceptance and completion.

### Limitations

This study has some limitations. First, the high treatment acceptance and completion rates reported in this study should be interpreted with caution, as participation was voluntary. This may have resulted in the inclusion of participants who were concerned about TB and motivated to take action. Furthermore, the treatment completion rates are largely based on self-reported data and corroborating community pharmacy data. As treatment was not always administered through directly observed therapy, there is potential for some inaccuracy in reported treatment completion. Second, while this study was designed to capture insights from immigrants across all immigration streams, most of the participants were refugees. As a result, we were unable to gather enough data from non-refugee immigrant groups, as initially intended. Future studies could consider using a stratified sampling design to overcome this limitation. Finally, the authors of this study acknowledge that the low sample size means that caution is required when interpreting the results and before any attempts to generalize to the population. The 49 individuals included here were captured from a larger sample of 264 screened individuals, making up 18.6% of that initial sample. Future studies should attempt to replicate these results using a larger sample size.

## Conclusion

In conclusion, this study achieved a treatment acceptance rate of 92.1% and a treatment completion rate of 85.7%, demonstrating the feasibility of addressing TBI in immigrants when healthcare teams work in partnership with local immigrant-serving organizations. Additionally, the study highlights several key factors that impact the management of TBI among immigrants in southern NB.

## Appendix

**Table A1:** Healthcare practitioner survey

1. What was your role in this study?	1. NP	2. ID Physician
2. Did you feel the patients were appropriately supported?	1. Yes	2. No
**Please explain:**		
3. Were there any barriers in supporting the patients during the initial visit?	1. Yes	2. No
**If yes, what were the barriers and how could they have been prevented?**		
4. Were there any barriers in supporting the patients during the subsequent visit?	1. Yes	2. No
**If yes, what were the barriers and how could they have been prevented?**		
5. Did you feel you had all the tools to care for the patients in your role?	1. Yes	2. No
**If no, what tools were missing?**		
6. Were there any barriers in arranging follow-up tests or visits?	1. Yes	2. No
**If yes, what were the barriers and how could they have been prevented?**		
7. What was helpful in caring for the patients?		
**Please state them below:**		
8. On average how long did you spend with each patient per visit (in hours)?		
**Please state them below:**		
9. How much time did you spend on patient management in total including administration, diagnostics, and arrangements?		
**Please state them below:**		
10. Do you have any final comments?	1. Yes	2. No
**If yes, please state them below:**		

Abbreviations: ID, infectious disease; NP, nurse practitioner
